# Abstracts

**Published:** 1918-08

**Authors:** 


### WAR MEDICINE ABSTRACTS

Information received from medical officers leads us to believe that the abstracts of original articles on war subjects published in the *Medical Bulletin* have made available to them a great deal of valuable material that has been helpful in their work of treating the sick and wounded soldiers of the A. E. F.

This review of the literature on subjects pertaining to the medicine, surgery, and hygiene of the war will continue to be an important feature of *War Medicine*.

It is not possible to publish an abstract of every valuable article appearing in the current medical literature of the war. Our effort, therefore, will be to select and review the most important contributions on these topics. These abstracts are not intended to be critical, but are supposed to present, impartially, the views of the authors of original articles, leaving to the reader the privilege of estimating for himself the value of the ideas contained in them.

The physicians with the American Expeditionary Forces have not access to medical libraries where they can get the journal containing any article which they see listed or reviewed; and on account of the delays and difficulties in transporting second-class mail, if they get medical journals at all, it is sometimes months after they are published; *War Medicine* is, therefore, the only medical publication which many receive. For this reason an effort is made to publish full abstracts, giving the essentials of the articles reviewed so that the complete articles will not be necessary, in most cases at least, to enable the reader to get the new and important facts brought out by the authors. However, if a medical officer desires to have the original of any article that is abstracted in *War Medicine*, the journal containing it will be sent him if he makes application to the Editor — provided, of course, that it has not already been sent to some one before the request is received. The Red Cross is establishing a Medical Information Bureau in connection with *War Medicine*, which, when organized, will be in a position to furnish any medical officer with all the literature available on any subject.

The abstracts published in *War Medicine* are not entirely the work of the editorial staff. Many of them have been contributed by a number of officers, who have voluntarily sent in reviews of what they considered important articles. For instance, in this number there are published a number of abstracts that were prepared by an American neurologist of the Medical Reserve Corps on duty in England; and a surgical team which happened to be in Paris during a lull at the Front, came to the A. R. C. Library and abstracted a number of surgical articles. While *War Medicine* is published by the American Red Cross in France it is for the American Expeditionary Forces; and American medical officers should feel that it is *their* medical publication, and that they can help make it of greater value to their medical compatriots by contributing abstracts of important articles that they read, or by making suggestions as to how *War Medicine* may be made of greater usefulness to them.

Time is an important element in giving to the medical profession the advances in medicine and surgery during the war; and American surgeons, when they have anything of value to publish, may get an abstract to their confreres through *War Medicine* several months before the original article could be published in a medical journal and returned to France; it is therefore suggested that, when they send to the Chief Surgeon the manuscripts of original articles to be published in medical journals, they also prepare and send abstracts giving the essential facts contained in them.

The Chief Surgeon of the A. E. F. is interested in having the medical officers receive the latest and best literature on all subjects pertaining to war medicine; and he has agreed to allow us to publish in *War Medicine* abstracts or preliminary notes of approved articles written by A. E. F. medical officers, which will later appear in medical journals. In publishing abstracts of articles not yet published, the title of the paper, with the author's name, will be given; and a note added stating that it is an abstract of, or a preliminary note on, an original article, the full text of which will appear later in a medical journal.

The heads of the professional services in the A. E. F. will be requested to designate medical officers to abstract the articles relating to the war that appear in certain journals. Since abstracts come from many sources and represent the collective effort of a large number of medical officers of the A. E. F., the names of the reviewers will not be published.

Many abstracts published in *War Medicine* no doubt seem elementary, and the information contained in them may not be new to the specialists in various lines; but it should be remembered that this publication of the Red Cross is intended especially for the medical officers who may not have access to any other medical publications, and who must treat all diseases, and wounds of every kind, that come to them. It is hoped, however, that each number will contain something of interest to every physician, surgeon, and specialist with the American Army in France.

